# Calibration of Failure Criteria for Additively Manufactured Metallic Materials

**DOI:** 10.3390/ma14133442

**Published:** 2021-06-22

**Authors:** Grzegorz Socha

**Affiliations:** Lukasiewicz Research Network—Institute of Aviation, Al. Krakowska 110/114, 02-256 Warsaw, Poland; grzegorz.socha@ilot.lukasiewicz.gov.pl

**Keywords:** failure criterion, calibration, additive manufacturing

## Abstract

A new version of failure criterion for additively manufactured materials, together with simple and accurate calibration procedures, is proposed and experimentally verified in this paper. The proposition is based on void growth-based ductile failure models. The failure criterion for ductile materials proposed by Hancock–Mackenzie was calibrated using simple methods and accessories. The calibration procedure allows the determination of failure strain under pure shear. The method is accurate and simple due to the fact that it prevents strain localization disturbing stress distribution at the failure zone. The original criterion was modified to better suit the deformation behavior of additively manufactured materials. Examples of calibration of the original and modified failure criteria for additively manufactured 316L alloy steel is also given in this paper, along with analyses of the obtained results.

## 1. Introduction

Additive manufacturing (AM) is gaining increasing importance as the aerospace component manufacturing technology. Despite significant progress in production technologies, additively manufactured components still cannot match the strength of precision casting, especially when they have to survive high service loads and high temperatures. On the other hand, AM components can offer some advantages; one of them being the absorption of impact energy. Due to an incomplete fusion of AM materials, such components, when subjected to compressive loads, can absorb significantly more energy in a smooth manner (avoiding peak loads that can be dangerous from the point of view of passenger safety). Properly designed AM manufactured energy-absorbing structures can offer some advantages compared to other manufacturing technologies, but the design and computer simulations of these deformation behaviors require use of appropriate yield conditions, flow rule and failure criteria.

As is widely accepted, failure criteria formulated in mixed strain and stress spaces are most suitable for ductile materials due to their sensitivity to the stress state. This sensitivity is even more pronounced in the case of additively manufactured materials. The effect of incomplete fusion on failure criteria of ductile materials can be easily deduced: failure strain will be much higher under compression than under tension. Some damage models based on void growth [1,2] allow the formulation of failure criteria in a mixed strain and stress space (mixed strain–stress criteria), such as the well-known criterion proposed by Johnson [3]. Such criteria can take this effect into account. In the case of Johnson criterion, applied in most popular finite element method codes (FEM) such as LS-DYNA, calibration coefficients can be found in the literature for popular alloys. For other criteria and alloys, calibration has to be performed if such criteria are to be used in computer simulations. 

Ductile failure models suitable for AM materials are based on void growth. In the case of incomplete fusion and the presence of physical discontinuities, void growth is believed to be most important damage mechanism. Among the most popular models of this kind are those proposed by Gurson [1] or Bai and Wierzbicki [2]. Such sophisticated models are popular among researchers, but, due to problems with experimental calibration, they are not popular in engineering and the material-testing community. Even most advanced criteria without credible calibration procedures are useless in engineering practice.

The credibility of the calibration method is of primary importance for the accuracy of computer simulation results. Experimental determination of equivalent plastic failure strain at a given stress state is a sophisticated and time-consuming procedure. An example of such a calibration can be found in [4] by Wierzbicki and colleagues. The main difficulty of failure strain determination is associated with the evolution of the specimen’s gauge part geometry and the localization of strain. This process is well known and has been investigated by many researchers. For example, Hutchinson and Nielsen investigated the formation of a cohesive zone (strain localization) for ductile metal plates [5]. Variations in the stress state of the neck of a tensile specimen has already been investigated in 1945 [6]. The obtained analytical solution of the boundary problem did not take into account strain hardening and its impact on the yield condition and flow rule. A more advanced approach based on slip line theory, accounting for strain hardening, was published much later [7]. Nowadays, sophisticated FEM computations are performed to investigate changes in the stress state at the gauge part of the specimen [4,5], but accurate determination of equivalent failure strains for a given stress state is still difficult due to problems associated with calibration of mathematical models applied to simulate deformation behavior of materials. 

In this paper, a calibration of failure criterion proposed by Hancock–Mackenzie [8] is presented. The applied calibration procedure requires no testing machine or specialized transducers. Another important advantage of the proposed method is fact that it allows to avoid strain localization. A modified version of the criterion, suitable for AM materials, is also proposed and calibrated using additional tensile tests.

## 2. Influence of the Stress State on Ductility

Triaxiality factor *η* was introduced into the failure criteria for ductile materials to take into account the sensitivity of the failure strain to the stress state. This sensitivity was proved at the beginning of the 20th century by the famous experiment of Von Karman [9]. His experiment showed that hydrostatic pressure (triaxial compression) significantly increases the ability of a material to achieve plastic strain preceding final fracture. 

Triaxiality factor *η* used in the failure criteria formulated in a mixed strain and stress space is given by the following formula:

(1) $$\eta =\frac{\sigma_{m}}{\sigma_{eq}}$$

where *σ_m_* stands for mean stress (hydrostatic part of stress tensor—average value of the three principal stresses) and *σ_eq_* stands for equivalent stress (some function of the second invariant of the stress tensor). In the case of Huber–Mises hypothesis, equivalent stress is given by the following formula:

(2) $$\sigma_{eq}=\sqrt{\frac{3}{2}I_{2}}$$

where *I*_2_ is the second invariant of stress deviator. 

The trouble with triaxiality factor *η* is that, for most experiments, including simple tension or compression, its value changes significantly due to the localization of strain before the final failure occurs. In the case of uniaxial tension of a round bar, this process is well known as “necking” of the gauge part of specimen and has been analyzed in many papers (among others, [6,7]). Knowing this experimentally proved fact, authors of [4] proposed procedure of averaging value of triaxiality factor for all the deformation process. The proposed average value of *η* for a given stress state is given by the following formula:

(3) $$\eta_{av}=\frac{1}{\varepsilon_{eq}^{f}}\int_{0}^{\varepsilon_{eq}^{f}}\eta \left(\varepsilon_{eq}\right)d\varepsilon_{eq}$$

where ε*_eq_* stands for equivalent strain. The integration of the equivalent strain increments along the deformation path in the strain space gives the result as a so-called Odquist parameter [10]:

(4) $$\varepsilon_{eq}=\int^{\;}d\varepsilon_{eq}$$

Final value of the Odquist parameter is the failure equivalent strain (final value of accumulated equivalent strain), and is denoted in Equation (3) as $\varepsilon_{eq}^{f}$.

The determination of the average value of triaxiality factor *η* for a given deformation process is an extremely complicated task, especially in the case of strain localization. For varying stress states, to calculate an equivalent strain increase, one has to use associated flow rule, initial yield surface, and its evolution in the stress space due to strain hardening. Using modern FEM software, such as LS-DYNA, it is possible to perform such a calculation [4,5], but to obtain credible results it is necessary to experimentally calibrate some strain-hardening models for the material in question. This may be a complex, time consuming, and expensive task. The experimental procedure used for calibration of strain-hardening model was described, for example, in [11]. In this case, complex stress was obtained as a result of simultaneous loading of tubular, thin walled specimens by axial load and torque. This technique allows the determination of the yield surface and its evolution resulting from the deformation history. The evolution of the yield locus can also be influenced by many phenomena, such as aging (also dynamic) or the strain rate effect [12,13]. Altogether, modeling of all the phenomena contributing deformation behavior can be an extremely complex task. 

Computer simulations of the strain localization can be regarded as a serious obstacle in the accurate determination of the average value of the triaxiality factor. Moreover, use of Equation (3) can be also questioned. Of course, the history of plastic deformation influences damage progress, but it is still uncertain how it influences failure strain in the final stress state. To bypass all the mentioned problems, a procedure and accessory allowing the calibration of mixed strain–stress failure criteria will be proposed. 

Criterion selected for calibration was given in [8] by Hancock and Mackenzie. The authors of this paper, on the base of an analysis of void growths, derived a failure criterion for ductile materials in the following form:

(5) $${\bar{\varepsilon }}_{f}=C_{1}exp\left(-\frac{3}{2}\eta \right)$$

where ${\bar{\varepsilon }}_{f}$ stands for the equivalent failure strain. This form of the criterion is suitable for an initially homogeneous material without voids or other flaws. As can be easily seen, only one test at a given stress state is necessary to calibrate this criterion (only one constant, *C*_1_, must be determined). This significantly simplifies the calibration procedure. 

The above-mentioned criterion (5) may be not accurate enough to predict the failure strain of AM materials. To account for the increased sensitivity of failure strain of AM materials to hydrostatic pressure a modified form of the failure criterion was proposed. For materials with voids and flaws that are due to incomplete fusion (such as in the case of AM materials) Criterion (5) can be modified as follows:

(6) $${\bar{\varepsilon }}_{f}=C_{1}exp\left(-C_{2}\eta \right)$$

Since the equation above includes a second constant, *C*_2_, calibration of this criterion requires at least two tests to be performed in different stress states.

## 3. Procedure and Equipment

As mentioned, only one constant (*C*_1_) must be determined to calibrate criterion (5). This criterion is easy to calibrate and accounts for the influence of the stress state on the failure strain. Bearing in mind all the mentioned difficulties described in [4], using the simple definition (5) and a more accurate and credible calibration procedure may be advantageous. An appropriate loading scheme must be selected to maintain the constant value of the stress triaxiality factor during the entire test. To avoid strain localization, it is very convenient to apply pure shear. 

**Figure 1:**
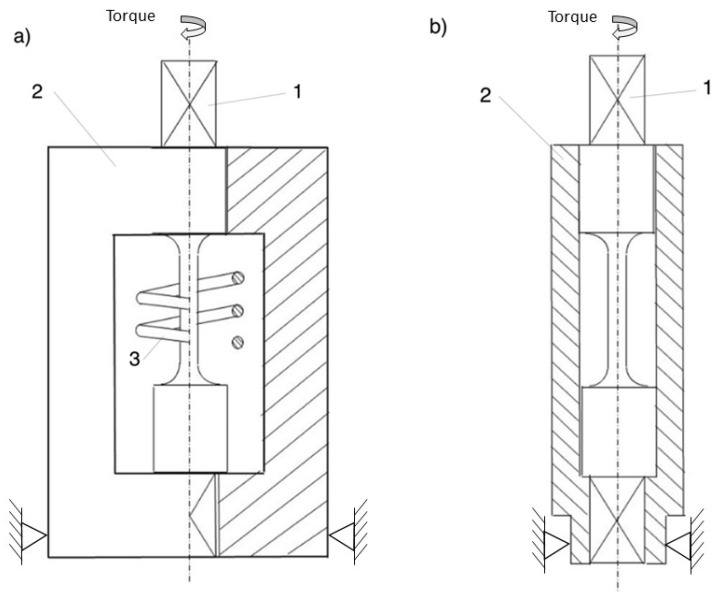
Two versions of setup for the determination of failure strain. Version (**a**) with an inductive heating coil (3) is suitable for elevated temperatures. Version (**b**) can be used for elevated, as well as lowered, temperatures.

Patent [14] described a method and accessories for the calibration of this simple failure criterion. To determine the failure-equivalent strain, we can use the setup shown in Figure 1a. The setup consists of a constrained yoke (2) with an inductive coil (3) for material heating. After inserting the specimen (1) into the yoke, inductive heating can start. When the desired temperature is achieved, the specimen is twisted to rupture using some kind of hand tool. An operator will try to maintain a constant rate of twisting and fit into the range of strain rates allowed by the appropriate standard. In the case of static tension “speed of testing when determining tensile strength” according to ASTM is between 0.05 and 0.5 l/min. For the investigated alloy, loading to rupture took time between 0.01 and 8 s. Since it is rather unlikely to twist specimen to rupture in less than 0.01 s using a hand tool, the operator, rather, needs to try to avoid too slow a deformation. For each material under consideration, the maximum time of the test should be roughly estimated using available data (ductility, standard elongation), and the applied rate of loading should allow to perform test in the allowable time limit range.

As a result of described procedure, the angle of twist of the gauge part is obtained. This twist angle can be easily re-calculated into a shear angle and then into failure shear strain (half of shear angle in radians). Finally, since loading is performed along a proportional path in the stress and strain space, an equivalent failure strain can easily be calculated using Formula (4), where integration can be omitted. 

Another version of the calibration setup is shown in Figure 1b. This kind of accessory is dedicated to calibration of the criterion at lower temperatures as well. A specimen (1) inserted in the sleeve (2) can be put into a cryogenic chamber to achieve the required test temperature. After the temperature is reached, the setup is moved from the chamber and clamped using a vice. Use of the sleeve allows stabilization of the temperature of the specimen gauge part for a period of time long enough to perform the test. As in the former case, the specimen can be twisted to rupture using a hand tool, removed from the sleeve and allowed to cool down. Measurements of the twist angle of the gauge part can be performed when the specimen reaches room temperature.

## 4. Calibration of Selected Criteria for 316L Alloy Steel

The specimen used for the test was additively manufactured from 316L alloy steel. Calibration of the failure criteria was performed at room temperature. The printing parameters were as follows: power of laser—180 W; distance between points—65 μm; exposition time—100 μs; speed—150 mm/s; spot size 55 μm; and the layer size—20–40 μm. Table 1 shows the chemical composition of the 316L alloy steel (in % of weight).

**Figure 2:**
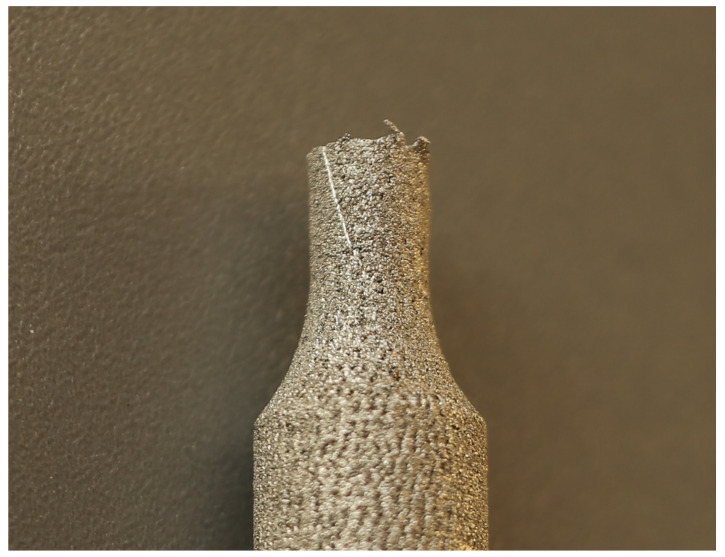
Specimen after the torsional test. Marked line, parallel to the specimen axis before test is inclined to specimen axis. Angle between axis and marked line is shear angle.

The broken specimen after the torsional test is shown in Figure 2. A line that is inclined at an angle to the specimen axis is visible in this figure. This line was drawn parallel to the specimen axis before the test. The strain localization, changing the stress state at the surface of the specimen, did not occur during the test, even when the twist angle of the gauge part reached its final value (corresponding to material failure). Stress triaxiality at the gauge part of the specimen was kept constant and uniform during all the deformation process. For applied pure shear, stress triaxiality factor *η* is equal to 0. Value of coefficient *C*_1_ is, in this case, equal to plastic strain intensity at failure. To obtain its value, maximum permanent (plastic) equivalent strain at the surface of the gauge part had to be found based on the angle of twist of the gauge part measured on microscope. For the geometry of the used specimen, shear angle was found to be *φ* = 0.14 rad. Based on this value and assuming proportional strain path in the strain space, the equivalent failure strain was found to be:

(7) $${\bar{\varepsilon }}_{f}=\frac{\sqrt{2}}{3}\sqrt{\frac{3}{2}\gamma^{2}\;}=0.08\;\mathrm{mm}/\mathrm{mm}$$

Introducing this value into Equation (5), failure criterion in the following form can be obtained:

(8) $${\bar{\varepsilon }}_{f}=0.08\;exp\left(-\frac{3}{2}\eta \right)$$

**Figure 3:**
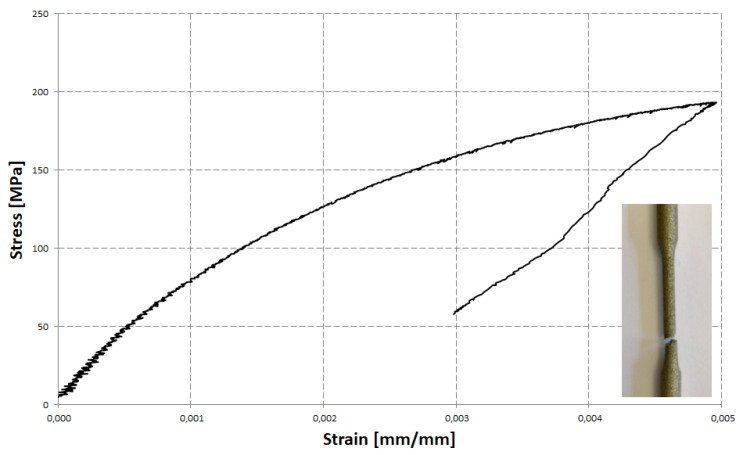
Tensile characteristics of 316L AM alloy steel with picture of broken specimen. Non-linearity of the characteristics is clearly visible.

As was mentioned, the criterion in this form (8) do not account for increased sensitivity of AM material to stress triaxiality. For this reason, a modified criterion in the form of Equation (6) was proposed. To calibrate this criterion, additional tests under another stress state needed to be performed. In Figure 3, the stress–strain curve, obtained as the result of static tension of a cylindrical specimen, is shown. The static tension test was performed according to the ASTM standard [15]. Strain in this case was measured with the use of an extensometer. Since failure of the specimen occurred outside the gauge part of the extensometer, the failure strain was found as the maximum strain before necking started. This conservative approach, disregarding localization of the strain and the formation of a cohesive zone was assumed to bypass all the complications associated with the determination of an average value of the triaxiality factor reported before. Since material can still survive some deformation, the failure strain is underestimated. Another, non-conservative approach is to use standard elongation of a tensile specimen. In this case the failure strain is overestimated due to the fact that strain localization contributes to the final value of the gauge length. Since standard elongation for many alloys can be found in the literature and Internet databases it makes calibration much easier (only one test is needed then).

The failure strain can be also calculated using the reduction of area [15]. This approach was used in another paper [16]. To calculate the failure strain intensity in the case of area reduction, we need to know all the components of the strain tensor for the narrowest cross-section of the specimen gauge part. This can be calculated using standard reduction of the area if we assume incompressibility. In the case of a void growth-based approach, this assumption cannot be accepted. In this paper, due to this fact and the very irregular fracture appearance (Figure 3), standard elongation was selected as the preferred approach.

Somewhere between the two mentioned solutions (conservative and non-conservative) is the exact solution. This solution is practically impossible to obtain due to the difficulties mentioned in previous paragraphs (calibration of material strain hardening the model in all the ranges of achievable strain in a complex stress state). Some approximation of the exact solution can be obtained using analytical [6,7] or numerical methods, such as FEM [5]. In the last case, the average value of stress triaxiality for the deformation process can be obtained using Formula (3). This approach, however, is the most advanced and is very elaborate; in the author’s opinion, the effect is not worth the effort. 

The specimen after the test is also shown in Figure 3. Non-linearity of the material characteristics from the very beginning of the application of load can be also seen in this figure. This non-linearity is the result of the stress concentration and the local yielding due to incomplete fusion of the AM material. As was mentioned, such a deformation behavior can be beneficial from the point of view of energy absorption because it allows avoiding peaks of load during a sudden crash of the structure. The maximum permanent uniform elongation measured by the extensometer was found to be 0.0027. Introducing this value, using the value of the stress triaxiality factor (*η* = 1/3) and the determined value of the coefficient *C*_1_ in Equation (6), the value of the second constant (*C*_2_) can be determined. For this conservative approach, a calibrated criterion takes the following form:

(9) $${\bar{\varepsilon }}_{f}=0.08\cdot exp\left(-10.16\cdot \eta \right)$$

The standard elongation for the material in question, determined according to ASTM [15], was found to be 0.004 mm/mm. For this non-conservative approach, the calibrated criterion takes the following form:

(10) $${\bar{\varepsilon }}_{f}=0.08\cdot exp\left(-8.98\cdot \eta \right)$$

**Figure 4:**
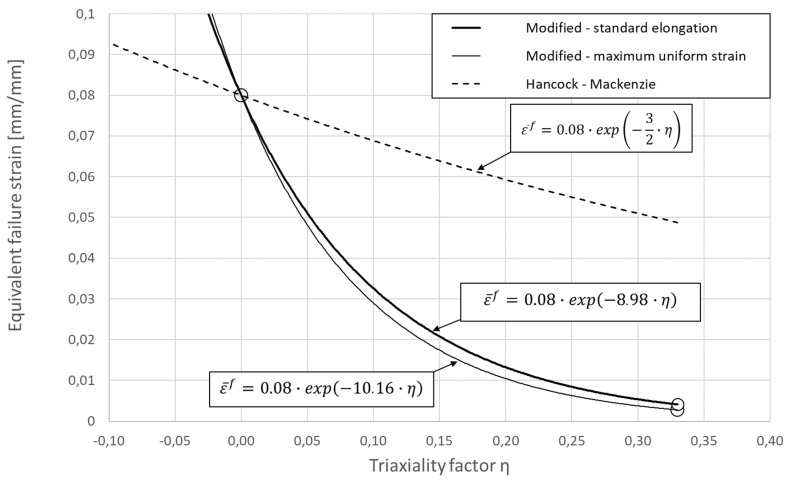
Calibrated criteria with graphical representation: Hancock–Mackenzie—broken line; modified criterion—solid line.

Both the calibrated criteria can be seen in Figure 4 in the form of plots, together with the corresponding equations. Experimentally determined points are marked with circles. The conservative approach is denoted by a thin line and the non-conservative by a thick line. As can be seen in the figure, the obtained solutions are quite similar to each other. For comparison, the Hancock–Mackenzie criterion is also shown in this figure as a broken line together with the corresponding equation. As can be seen, the modified criterion is more sensitive to stress triaxiality; which is, in the case of AM materials, in the agreement with the intuition and initial assumptions.

## 5. Summary and Conclusions

A new version of failure criterion for additively manufactured materials, together with a simple and accurate calibration procedure, was proposed and experimentally verified. The simple and accurate calibration procedure is crucial for applicability and credibility for any failure criterion. 

Calibration of the failure criteria for ductile materials is a time consuming and sophisticated procedure. The main difficulty of the procedure is associated with strain localization changing the stress state at the gauge part of a specimen. Patent pending fixtures [14] allow the determination of failure strain intensity at pure shear, avoiding strain localization. For 316L AM alloy steel, the proposed procedure allowed the determination of equivalent failure strain without using a testing machine equipped with specialized transducers. The test was performed with a constant value of stress triaxiality factor, from the beginning of deformation to the final failure of the material. 

Modification of Hancock–Mackenzie failure criterion was proposed for AM materials. The modified criterion can be calibrated with the use of the proposed procedure and additional tests, performed under another stress state. In the case of the presented investigations, uniaxial tension was used as the additional test. Disregarding strain localization, we can use the maximum uniform strain of the gauge part, measured by an extensometer to calculate the equivalent failure strain for the corresponding stress triaxiality factor. This approach, while conservative (failure strain is underestimated), seems to be proper from the point of view of safety. 

Alternatively, standard elongation of the specimen’s gauge part to calculate failure strain and obtain a non-conservative approach can be used. Between the obtained two curves shown in Figure 4, the exact solution is located. The exact solution using an analytical or numerical approach is possible to obtain, but then averaged value of triaxiality factor has to be used. Both mentioned solutions require the use of a calibrated model of strain-hardening for the material in question, which is hardly available and requires complex stress testing. 

Despite all the mentioned difficulties, the tensile test enables determination of the second constant of the modified failure criterion (6). Since standard elongation for many alloys can be found in the literature and in Internet databases, it makes calibration much easier. In this case, only one torsional test is needed to calibrate the modified criterion. This modified criterion is more appropriate for AM materials since it can better reflect its increased sensitivity to the stress state.

## Figures and Tables

**Table 1:** Chemical composition of 316L alloy steel.

Cr	Ni	Mo	C	Mn	Cu	P	S	Si	N	Fe
17–19	13–15	2.25–3	0.03	2.0	0.5	0.025	0.01	0.75	0.1	balance
